# The need for digital health education among next-generation health workers in China: a cross-sectional survey on digital health education

**DOI:** 10.1186/s12909-023-04407-w

**Published:** 2023-07-31

**Authors:** Mingxue Ma, Yuanheng Li, Lei Gao, Yuzhuo Xie, Yuwei Zhang, Yazhou Wang, Lu Zhao, Xinyan Liu, Deyou Jiang, Chao Fan, Yushu Wang, Isaac Demuyakor, Mingli Jiao, Ye Li

**Affiliations:** 1 Harbin Medical University, 157 Baojian Road, Nangang District, Harbin, 150086 Heilongjiang China; 2 Heilongjiang University of Traditional Chinese Medicine, 24 Heping Road, Xiangfang District, Harbin, 150006 Heilongjiang China

**Keywords:** Next generation doctors, Medical education, Digital health, Cross-sectional survey, Curriculum design, Chinese medical students, Students as partners

## Abstract

**Background:**

Digital health is important for sustainable health systems and universal health coverage. Since the outbreak of COVID-19, many countries, including China, have promoted the introduction of digital health in their medical services. Developing the next generation of physicians with digital health knowledge and skills is a prerequisite for maximizing the potential of digital health.

**Objective:**

We aimed to understand the perception of digital health among Chinese medical students, the current implementation of digital health education in China, and the urgent need of medical students.

**Methods:**

Our cross-sectional survey was conducted online and anonymously among current medical students in China. We used descriptive statistical analysis to examine participant demographic characteristics and the demand for digital health education. Additional analysis was conducted by grouping responses by current participation in a digital health course.

**Results:**

A total of 2122 valid responses were received from 467 medical schools. Most medical students had positive expectations that digital health will change the future of medicine. Compared with wearable devices (85.53%), telemedicine (84.16%), and medical big data (86.38%), fewer respondents believed in the benefits of clinical decision support systems (CDSS) (63.81%). Most respondents said they urgently needed digital health knowledge and skills, and the teaching method of practical training and internship (78.02%) was more popular than the traditional lecture (10.54%). However, only 41.45% wanted to learn about the ethical and legal issues surrounding digital health.

**Conclusions:**

Our study shows that the current needs of Chinese medical students for digital health education remain unmet. A national initiative on digital health education, is necessary and attention should be paid to digital health equity and education globally, focusing on CDSS and artificial intelligence. Ethics knowledge must also be included in medical curriculum. Students as Partners (SAP) is a promising approach for designing digital health courses.

## Introduction

### Background

Presently, healthcare delivery faces a high burden of infections and non-communicable diseases, dearth of human resources, inequitable distribution of healthcare, lack of personalized care, and limited preparedness for emergencies in both developed and underdeveloped countries [1]. World Health Organization (WHO) considers digital health a key driver in addressing these health challenges and achieving sustainable health systems and universal health coverage. It has identified priorities for digital health strategy in the Global Strategy Report for Digital Health (2020–2025) [2]. The benefits of digital health include integrating data across services, providing electronic decision support, resources, and interventions, improving patient-physician communication, and developing digital devices that facilitate monitoring and positive behavior change [3]. Digital health technology achievements have fundamentally changed how and where healthcare is delivered, including how it is organized, as it facilitates health monitoring, improves the quality of life outside of traditional healthcare attitudes, and goes hand in hand with the public’s hope for better and more effective patient care [4–7]. Especially during the coronavirus disease (COVID-19) crisis, digital health has been recognized as an innovative health solution that ensures continued access to clinical care and enables public health action to stop the rapid spread of the virus and accelerate the implementation of digital health during the pandemic [8–10]. The global epidemic of infectious diseases is still very serious and recurring. Digital technologies such as artificial intelligence will be assigned new tasks and directions for normalized prevention and control of infectious cases in the post-epidemic era.

Adopting appropriate digital health technologies and exploring the potential of global solutions and shared services have been identified as key components of national health strategies [2]. Work is underway worldwide to promote the adoption of digital health in health services. Governments in the United States, United Kingdom, France, India, Argentina, and other countries have also made important policy changes to encourage and incentivize the use of digital health [1, 11–13]. University of Toronto is trying to integrate neural networks into massive data sets from hospitals around Toronto and is working on using artificial intelligence to analyze genomes. These are just a few of Canada’s digital health initiatives [14].

As the largest developing country, China has a relatively inadequate supply of high-quality and unevenly distributed medical resources. Digital health is particularly important for improving the existing medical conditions in China. By the end of October 2020, there were 900 internet hospitals in China, and the telemedicine collaboration network included more than 24,000 medical facilities [15]. In the post-pandemic period, the Chinese government’s support for digital health has been strengthened and Chinese digital health industry has also experienced rapid development. CB Insights published the Global Top 150 List of Digital Health Companies in 2020, which lists 7 Chinese digital health startups, ranking second worldwide after the United States. According to some studies [16–18], China is expected to lead in AI medicine because of its unique data, government support, investment from venture capital funds, participation from top universities, and a very favorable regulatory environment. However, there are still significant gaps in digital health in resource-limited settings, and technical and sociocultural differences exist between different regions or between provinces in the same region [19]. While the implementation of digital health has been very heterogeneous, the potential of digital health is enormous [20].

Progress toward the widespread and sustainable adoption of digital technologies in specific clinical settings and health systems worldwide is still relatively slow [21]. The lack of knowledge and awareness about new technologies and the skills to use them among health professionals is one of the major barriers to the application of digital health in clinical practice [22]. In the Global Digital Health Strategy 2020–2025, WHO explicitly proposes to integrate knowledge and skills related to digital health into the education and training curricula of healthcare and allied health professionals [2]. Youth’s high levels of digital engagement and literacy skills put them in an excellent position to understand the fundamental requirements for successful digital health implementation [23]. It is important to educate future healthcare professionals about current and foreseeable technological innovations and enable them to adapt to future changes in their field [24–26]. However, given the limited digitization of learning health management systems, concerns remain about privacy, security, quality, and accuracy in medical education [27]. Therefore, there is an urgent need to establish systematic courses on digital health in medical schools to train the next generation of doctors in the integration of medical theory and digital technology and equip them with the ability to work in the digital medical system in the future [4].

Currently, many universities and research institutions have recognized the importance of increasing the digital health literacy of the next generation of physicians. The number of medical schools introducing digital health courses and teaching are increasing internationally. For example, Harvard University, Brown University, the University of Queensland, the University of Berlin, Freie Universität Berlin, the European Medical Students’ Association (EMSA), and other research institutions have conducted relevant studies and research, as well as the design and implementation of digital health courses [28–31]. However, there are still relatively few studies on the cultivation of digital health ability of next generation of doctors in China. To the best of our knowledge, this study is the first to investigate the awareness of digital health and the demand for digital health education among medical students in China on a wide scale.

### Aim

Our goal was to understand the perception of digital health among Chinese medical students, and the realistic demand for digital health-related knowledge and skills, and fill the gap in the research on digital health courses for medical students in developing countries. We sought answers to the following questions:

1. How do medical students view digital health and its future?
2. What are the practical problems associated with providing digital health education in China?
3. What kinds of knowledge and skills do medical students want to acquire about digital health and how will it be delivered?

## Methods

### Study design and recruitment

The first draft of the survey questions was developed during four online discussions after conducting necessary literature research and receiving feedback from experts in digital health and medical education. To confirm the effectiveness, clarity, readability, accessibility, and functioning, pilot surveys were conducted on 239 medical students. The data obtained were not used for the final analysis. We developed 46 questions that included the following parts: (1) sociodemographic information; (2) digital health knowledge survey; (3) digital health education needs survey.

From April 12 to May 10, 2022, an online cross-sectional survey was conducted among a nationwide population of medical students in China. Participants were recruited online. At the beginning of the online survey, it was stated that submission of the online questionnaire meant that the participant consented to participate in the study. The responses to the online questionnaire were anonymous and did not ask for personal information such as name or email address. The survey was emailed to 2891 individuals within 30 days of recruitment, and a total of 2122 (73.4%) valid responses were received.

To ensure that the data collected have high quality, we conducted reliability and validity analyses of the questionnaire. Reliability reflects data stability and concentration. We tested the reliability of the scales used in this study. The Cronbach’s alpha of this study was 0.722 (> 0.7), indicating acceptable reliability and consistency of the data. According to the result of the validity analysis, the value of the Kaiser–Meyer–Olkin was 0.862, the significance probability of the Chi-square value for Bartlett’s Test of sphericity was less than 0.05, and the questionnaire had good coverage and scientific nature. The design of the questionnaire was checked by experts in the field, and its good content validity was proven.

### Statistical analysis

SPSS Statistics 25 (IBM Corp) was used for statistical analysis. Descriptive statistical analysis was used to examine participant demographic characteristics and their needs for digital health education. Based on their answers to the question, “Are you currently taking a digital health course at university?” we divided the students into two groups – yes or no – and the χ2 test was used to compare the rates, P value less than 0.05 was considered statistically significant for questions with possible answers ranging from 0 (strongly disagree) to 6 (strongly agree). The options “undecided” and “I feel under-informed” were placed next to each other in the linear regression model, because they were halfway between the extreme values of 0 and 6.

## Results

### Demographic data

This survey was screened for validity and completeness. For example, > 80% of the questions showed monotonous response patterns, and a total of 2122 participants were finally included in this study. 1043 of the participants in our study were male and 1079 were female. The number of medical students in the fifth grade and above is relatively small (150/2122, 7.06%), because in China medical students finish their schooling in the fourth year of professional training and go to the hospital for internship. The majority of respondents (all respondents are older than 16) were aged between 18 and 22 (1674/2122, 78.89%), followed by 23 years or older (448/2122, 21.11%). Respondents came from 467 colleges and universities across the country, with the largest number of students whose schools were in Beijing (287/2122, 13.52%), followed by those whose schools were in Guangdong (221/2122, 10.41%) (Table 1).

**Table 1:** Descriptive statistics of respondents

Demographic Variables	Subgroups	Frequency (*N* = 2122)	Percentage (100%)
**Gender**	Male	1043	49.15
	Female	1079	50.85
**Age (years)**	≤ 18	44	2.07
	18–22	1674	78.89
	≥ 23	448	21.11
**Region**	East	1245	58.67
	Middle	524	24.69
	West	353	16.64
**Professional**	Clinical	945	44.53
	Non-clinical	1177	55.47
**Grade**	1	203	9.57
	2	704	33.18
	3	765	36.05
	4	299	14.09
	5	48	2.26
	> 5	103	4.85

### Digital health awareness

**Fig. 1:**
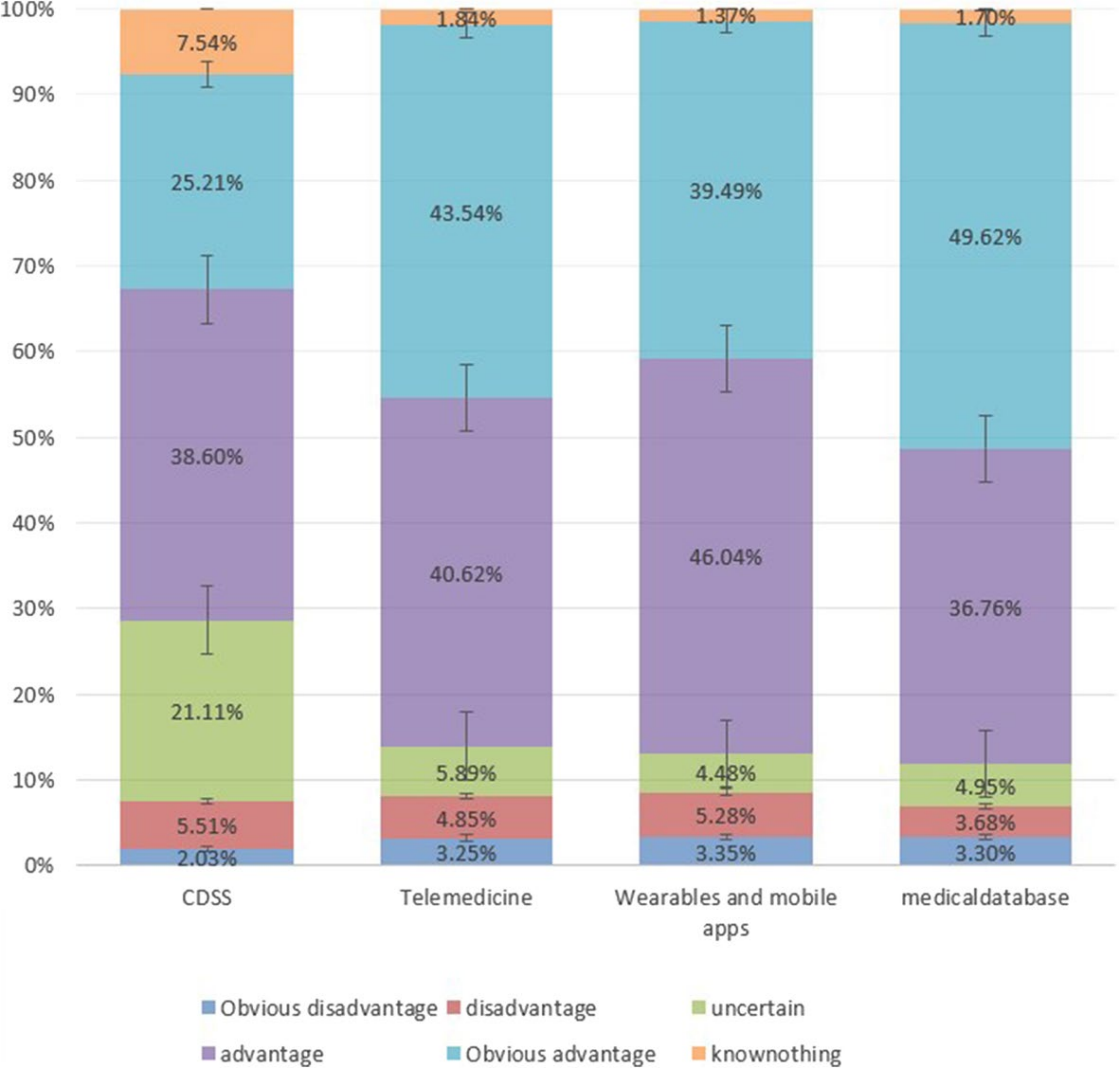
Students’ perception of the future use of digital health technologies

Our survey shows that 23.52% of respondents are unfamiliar with or unsure about digital health, and 13.2% do not know the definition of digital health. Regarding the daily use of digital health technology, 60.23% of respondents use health apps or wearable devices more than three days a week. 5.94% of respondents have never used digital health technology. Overall, medical students have positive expectations that digital health will change the future of medicine. More than 80% of the respondents believe that the future use of wearable devices and mobile apps and the future use of telemedicine is advantageous. Most respondents (86.38%) believe that the use of Big Medical Data will bring benefits in the future. However, only 63.81% of respondents consider it beneficial to rely on CDSS for treatment/diagnosis/analysis and data ranking (Fig. 1).

**Fig. 2:**
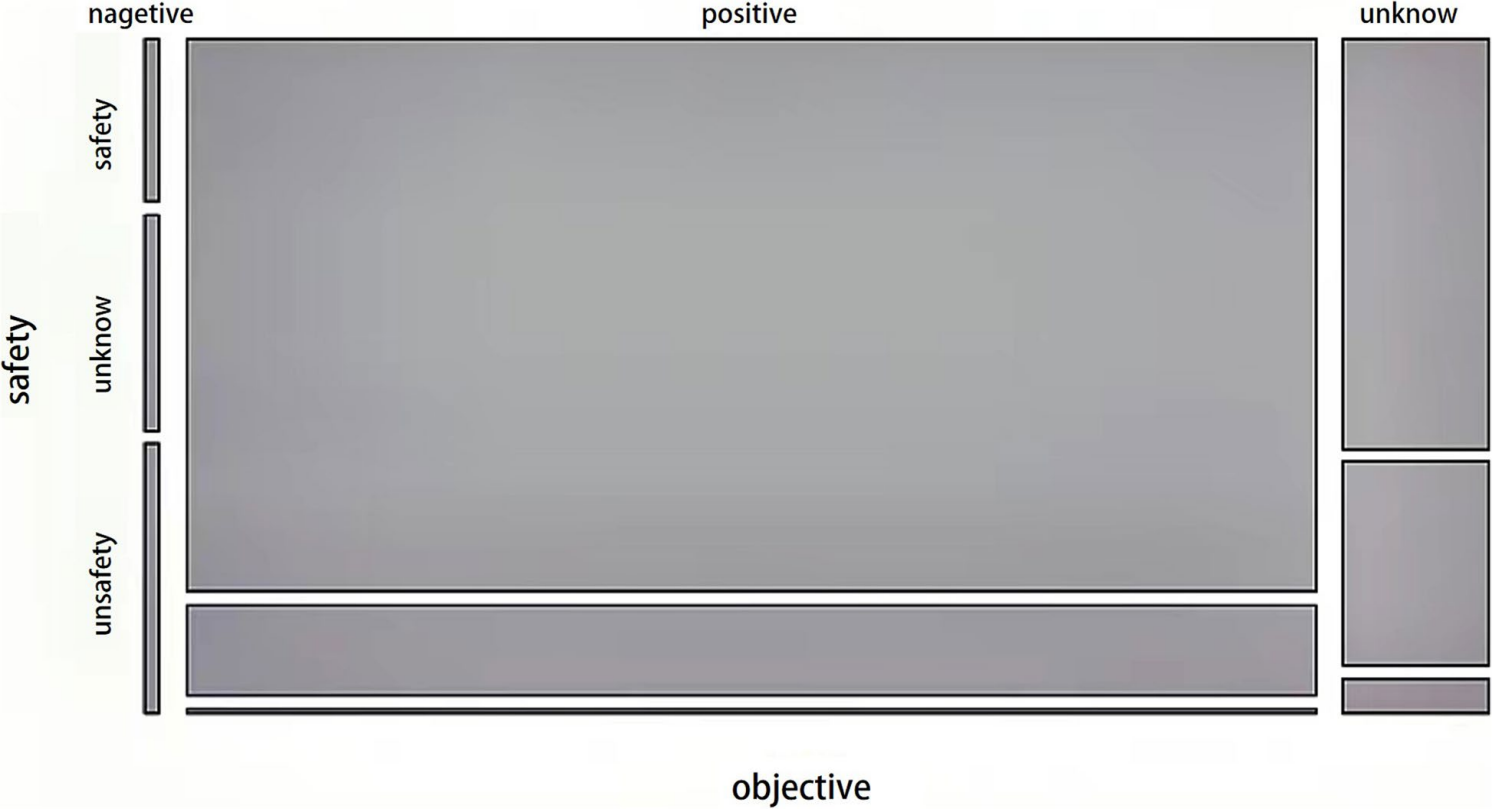
Perspectives on the future application of digital technologies to medical practice and research

More than 80% of respondents believe that the future application of digital technologies in medical practice and research is safe. 16.21% of respondents are not sure about the future use of digital health technologies, and 1.74% think it is unsafe or very unsafe (Fig. 2). Our survey also found that respondents who had taken a digital health course in college were more familiar with digital health terminology (*P* < 0.001) and were more confident and optimistic about the future use of digital technologies in medical practice and research than those who had not (*P* < 0.001) (Table 2).

**Table 2:** Whether the respondents received or received digital health course training and their awareness of digital health

Variables	Does your school offer digital health related courses		
	t	P>|t|	[95% Conf. Interval]
I’m familiar with the term ‘digital health.‘	-15.85	0.000	− 0.3218175 − 0.2509545
Digital health will change the future of medicine	-4.80	0.000	− 0.0995811 − 0.0417743
The security of digital technology	-9.98	0.000	− 0.246377 − 0.1654574
Use the optimism of digital health	-9.82	0.000	− 0.2145355 − 0.1431269

### Digital health course needs

According to the survey, 87.24% of respondents believe that it is necessary to prepare to enter the digital health system. More than 90% of the respondents report that there is an urgent need to acquire knowledge and skills in digital health. Most respondents’ schools offer digital health courses, and there was a statistical difference between the schools in the East, middle and West (*P* < 0.05) (Table 3). The use of digital health technologies accounts for 78.43% of the digital health courses offered by schools, while the ethical issues and legal knowledge related to digital health account for less than 60%. Approximately 56.97% of respondents have taken a digital health course, of which only 20.26% have spent more than 40 h and the majority (53.35%) have spent less than 20 h. A rate of 62.87% of respondents plan to take self-study digital health courses, and 29.9% have taken or are taking self-study digital health courses.

**Table 3:** Whether the respondents’ schools offer digital health related courses

Region	Total N (%)	Have you currently taken a digital health course at your university?			*p*-value
		Yes	No	I don’t know	
East	1245 (58.67%)	793 (63.69%)	321(25.78%)	131 (10.52%)	0.023*
Middle	524 (24.69%)	306 (58.40%)	158(30.15%)	60 (11.45%)	
West	353 (16.64%)	204 (57,79%)	94(26.63%)	55 (15.58%)	

0.01< *P < 0.05

**Fig. 3:**
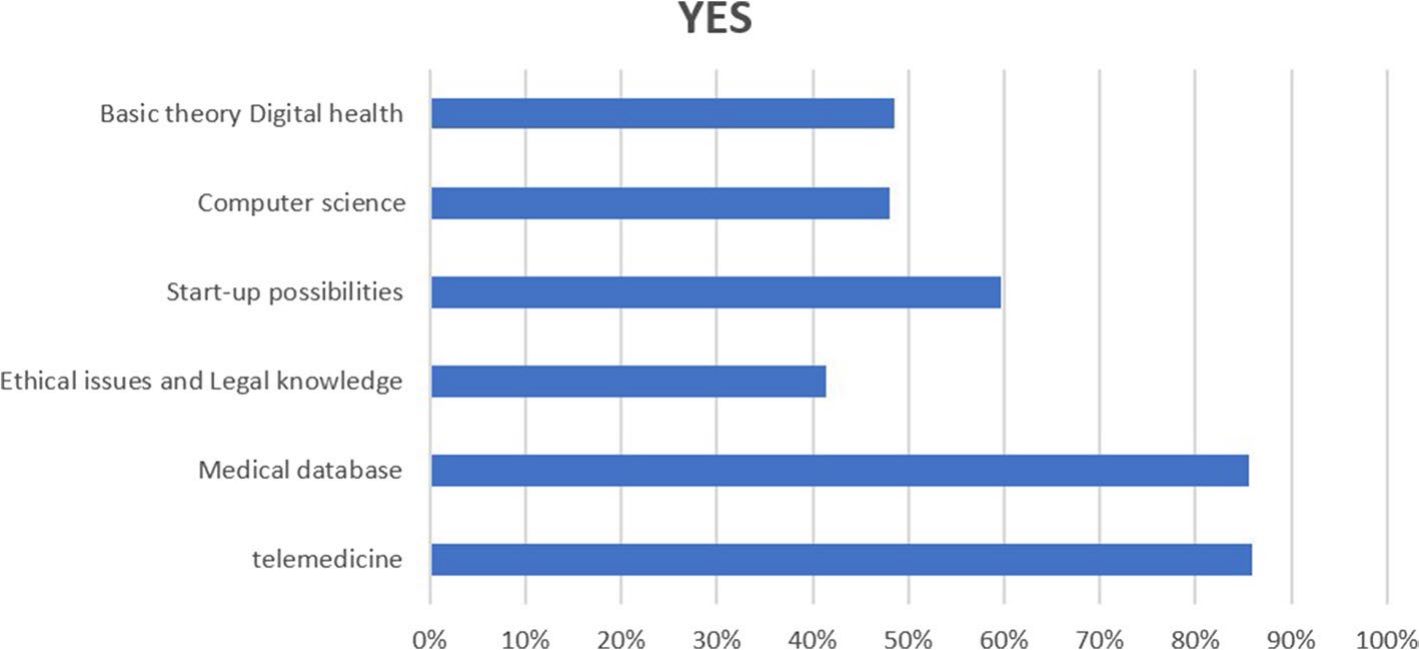
An overview of the digital health-related topics respondents expect to learn

**Fig. 4:**
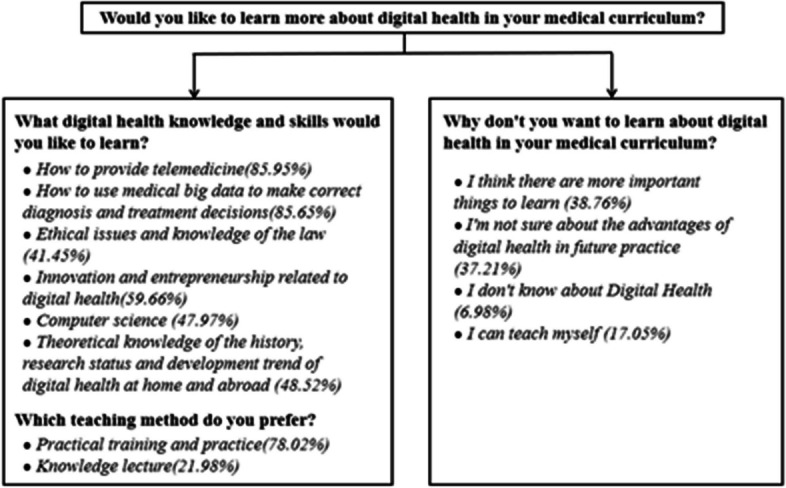
Respondents’ reasons to want or not want to learn the content of digital health courses

More than 80% of the respondents want to learn more about digital health in medical courses; 85.95% prefer to learn how to provide telemedicine; 85.65% prefer to learn how to use big medical data to correct diagnoses and treatment decisions. However, only 41.45% of respondents want to learn about the ethical issues and legal knowledge of digital health (Fig. 3). Respondents are more inclined to make digital health a required subject than an elective one. The teaching method of practical on-the-job training and internship (78.02%) is more popular than traditional lectures (10.54%). A total of 129 respondents (6.08%) do not want to learn about digital health; 37.98% of them think they need to learn more; 36.43% of people are not sure about the advantages of digital health practices in the future and do not want to learn digital health; 17.05% of participants think that this content can be self-taught (Fig. 4).

## Discussion

### The need for digital health education

The benefits of digital technologies as important prerequisites for sustainable health systems and universal health coverage have already been highlighted, especially during COVID-19 [2, 32]. As the next generation of physicians, medical students need to have the knowledge and practical skills in digital health that are relevant to their future practice [29]. As the most digitally literate generation to date, contemporary medical students are in a unique position to effectively implement the digital transformation of the health sector. Our study shows that current medical students have a high awareness of digital health and that the use of digital health technologies is common in daily life. Medical students are full of expectations about the future of digital health and believe that digital health technologies have obvious advantages for, and are likely to revolutionize the future of medicine. These factors increase medical students’ willingness to engage in digital health. However, a European survey showed a gap between students’ overall readiness to become key players in the meaningful digitization of healthcare and the competencies and skills they have acquired through their learning [30]. Our study also proves the urgent need to fully integrate digital health into the curriculum to provide digital health education to medical students, as more than 80% of respondents would like to learn more about digital health in their medical curriculum. Due to the lack of digital health education for medical students, many have begun to increase their digital health knowledge and skills through self-study.

### The development and popularization of digital health technology

Our study shows that current medical students are positive about the benefits of the future use of wearable devices and mobile apps, telemedicine, and medical big data. However, there are still some concerns about the decision to rely on CDSS for treatment/diagnosis/analysis and data ranking. CDSS generally refers to a computer system that provides auxiliary support for clinical medical decision-making based on an artificial intelligence deep learning algorithm. A systematic review shows that CDSS can improve the quality of clinical decision-making and healthcare processes as well as patient outcomes [33]. The implementation of CDSS involves significant resources and expenditures; however, several studies have shown that the use of these tools in clinical practice is generally low due to several factors [34–37]. Whether clinicians have used CDSS during their studies has an important impact on the willingness of medical staff to use CDSS [38]. CDSS is only one of the early stages of medical AI. Soon, physicians will accept AI and algorithms as their working partners, a culmination that Eric Topol calls “deep medicine” [18], whereby deep learning – a type of machine learning – is applied to the medical field. Therefore, it is critical for the next generation of physicians to strengthen education and training related to CDSS and AI to ensure they can use AI as a tool in the future. In addition, when educating medical students in digital health, we can also seek to cultivate their ability to participate in the development and achieve interdisciplinary integration with computer science and finance to provide medical students with a broader development space.

### Equity in digital health education

Digital health courses driven by a single pilot project adapted to the individual curricular conditions of medical faculties are trickling into the medical curriculum [28, 39–43]. It can be the first step toward realizing a longitudinal interdisciplinary approach to implementing digital health in the curriculum [28, 44–47]. Our survey found that more than half of the respondents had already taken courses on digital health offered by their medical schools. However, existing research demonstrated a regional imbalance in education in China [48]. To some extent, the survey results also indicated a regional imbalance in China’s digital health education. Su revealed that the development level of the digital economy in the eastern region of China is significantly higher than that of the whole country and the other regions [49]. This is similar to our research results. In the economically developed eastern region of China, more medical students are receiving digital health training. However, in western China, where the economy is relatively underdeveloped, many medical students are improving their digital health expertise through self-study because their educational needs are not being met. The lack of coordinated, formal education on the use of digital technologies in health is one of the main factors limiting the readiness of current and future health professionals for digital health [48]. Studies have shown that the next generation of health professionals can better address and prevent issues such as disparate use of digital health technologies [20, 49]. We still require a national initiative, to help support the adoption of a systematic approach to curriculum design, via collaboration with various stakeholders, to enable the much-needed transformation of digital health [48, 50]. These findings also suggest that we should be mindful of long-term sustainability and equity in the global adoption of digital health technologies and digital health education and training.

### Research on digital health courses

The global shortage of health workers has been identified as a major barrier to achieving universal health coverage [51, 52]. Digital health education has been identified as a potential means to address these growing challenges [53]. A major shortcoming is the lack of research relevant to a digital health curriculum [29].

The ethical issues around new technologies have always attracted much attention both in China and internationally. With the rapid development of digital health worldwide, it is becoming increasingly important to pay attention to the ethical issues related to the application of digital technologies to health [54–56]. A strong focus on training on “governance, quality, security, standards, privacy, and data ownership,” as highlighted by WHO, will be a key feature of a successful “meaningful digital health connection” [24]. However, our study found that respondents’ awareness of ethical issues and legal knowledge related to digital health is significantly lower than their knowledge of clinical practice and application. The Council of International Medical Organization and WHO have published research which demonstrates that the international ethical guidelines for human health follow the principle of medical ethics in detail, but the ethical framework applied to digital health is still new and comes from medicine, economics, computer science, social science, law, and investment decision-making [24, 55–58]. For the next generation of physicians who will work in digital medicine, acquiring the ethical knowledge reflected by artificial intelligence and other relevant digital technologies is an extremely important part of their medical curriculum [59, 60] that should be given attention early in their engagement with digital health technologies. In designing digital health training courses, in addition to considering students’ actual needs in terms of clinical practice and digital health application skills, ethics courses related to digital health should be included to meet the needs of the next generation of physicians and improve their medical humanistic competence [8].

Our results also suggest that medical students prefer hands-on training and practice to the passive knowledge transfer of lectures. This finding provides a new vision for digital health curriculum design for medical students. The way in which student preferences are incorporated into the design and delivery of educational programs benefits both staff and students, as well as the institution, and enhances the higher education experience and learning [61–63]. Digital health as an emerging discipline in medical practice makes students key stakeholders in curriculum development, which will help identify learning aspects intrinsically linked to students’ professional practice [29]. The Students as Partners (SaP) initiative has gained increasing attention over the past decade. Many institutions praise the SaP model as a way to enhance collaboration, reciprocity, and peer learning [64]. Co-creation of learning and teaching occurs when staff and students work together to create curriculum and/or pedagogical approaches [65]. Participatory action research has been discussed in depth in chemical engineering and other fields and has produced amazing results [66]. Student partners were constructively involved in the course design process, which added significant value to the redesign of the teaching modules and generated a real response from fellow students. We believe that this approach is a good reference for digital health course designers.

In addition, our study found that medical students want digital health to become a required course in the medical profession because it is significant for the future development of medicine. Some studies show that digital health should best be taught in the early part of the medical curriculum (e.g., year 1) and that digital health practice and behaviors and clinical applications should be taught in the final year of medical education [29, 67]. Furthermore, the results of this study show that there is a need to integrate the teaching of digital health with other specialized courses and interdisciplinary development. This is consistent with a European survey on perceptions of digital health teaching among medical students [30].

### Limitations

Based on a cross-sectional survey of 2122 medical students from 467 medical schools nationwide on their perceptions and expectations of digital health education, the results show that medical students have a high awareness of digital health knowledge and skills and are particularly supportive of their schools offering lectures/courses on digital health knowledge and skills. However, our study has some limitations.

As our study conducted an online cross-sectional survey, a large sample size was considered; hence, it only reflects the subjective views of the medical students surveyed. Furthermore, this affected the reliability of the survey results and the accuracy of the samples to some extent. To obtain more objective results, a qualitative interview study on digital health education of medical students will be conducted in the future.

The idea that there lies inequality in the provision of digital health education lacks support from the data available at the school level. In future, we will conduct a sample survey of the digital health courses offered by medical schools across the country.

In addition, the survey object of this study is limited to medical students and does not cover all stakeholders, so it is not representative of all relevant parties. Therefore, a larger sample population will be needed in the following study to better develop the implementation plan for digital health education.

## Conclusions

Our study was an online cross-sectional survey regarding the awareness of and accessibility to digital health education among Chinese medical students. We believe that the current emphasis on digital health education needs improvement, especially in the field of AI such as CDSS; in the construction of digital health curriculum system in the future, more attention should be paid to the teaching of specific use knowledge of digital health and the training of practical operation skills; more attention should be paid to the ethical and legal knowledge related to digital health, and relevant theoretical research still needs to be followed up with the development and popularization of digital health technology. In addition, the ability of medical students to participate in the development of digital health technologies should be encouraged, and interdisciplinary integration with computer science and finance should be utilized to provide medical students with a broader development space.

Our study also found that inequity in digital health education has emerged, which suggests that the long-term sustainability and equity of digital health education on a global scale should be addressed. International and standardized digital health education initiatives are needed to ensure the digital health skills of current and future healthcare workers are kept up to date and to the expected standard. In addition, our study highlights that students are a valuable resource and should be key stakeholders in the development of digital health curricula, and that the SaP approach is a good reference for the curriculum developers.

## Data Availability

The datasets used and/or analysed during the current study are available from the corresponding author on reasonable request.

## Abbreviations

CDSS: Clinical decision support systems
WHO: World Health Organization
COVID-19: Coronavirus disease crisis
EMSA: European Medical Students’ Association
AI: Artificial intelligence
