# Correction: The need for digital health education among next-generation health workers in China: a cross-sectional survey on digital health education

**DOI:** 10.1186/s12909-023-04613-6

**Published:** 2023-09-22

**Authors:** Mingxue Ma, Yuanheng Li, Lei Gao, Yuzhuo Xie, Yuwei Zhang, Yazhou Wang, Lu Zhao, Xinyan Liu, Deyou Jiang, Chao Fan, Yushu Wang, Isaac Demuyakor, Mingli Jiao, Ye Li

**Affiliations:** 1 Harbin Medical University, 157 Baojian Road, Nangang District, Harbin, 150086 Heilongjiang China; 2 Heilongjiang University of Traditional Chinese Medicine, 24 Heping Road, Xiangfang District, Harbin, 150006 Heilongjiang China

Correction: BMC Medical Education (2023) 23:541

10.1186/s12909-023-04407-w

Following publication of the original article [1], the authors informed us that the order of the figures is wrong. The content of Fig. 1 should be placed in the position of Fig. 2, the content of Fig. 2 should be placed in the position of Fig. 4, the content of Fig. 3 should be placed in the position of Fig. 1, and the content of Fig. 4 should be placed in the position of Fig. 3

The original article has been corrected.
